# Prepregnancy Diet Quality Index and Adverse Pregnancy Outcomes

**DOI:** 10.1001/jamanetworkopen.2026.33245

**Published:** 2026-09-10

**Authors:** Ummayhany Bharmal, George Saade, Philip Greenland, Brenda J. Smith, Andrea C. Kozai, Stephanie A. Fisher, Lauren Theilen, William Grobman, C. Noel Bairey Merz, Uma Reddy, Tetsuya Kawakita, MS

**Affiliations:** 1 Department of Obstetrics and Gynecology, Medical College Baroda, Vadodara, Gujarat, India; 2 Department of Obstetrics and Gynecology, Macon and Joan Brock Virginia Health Sciences at ODU, Norfolk, Virginia; 3 Departments of Preventive Medicine and Medicine, Northwestern University Feinberg School of Medicine, Chicago, Illinois; 4 Department of Obstetrics and Gynecology, Indiana University School of Medicine, Indianapolis; 5 Department of Epidemiology, School of Public Health, University of Pittsburgh, Pittsburgh, Pennsylvania; 6 Department of Obstetrics and Gynecology, Northwestern University Feinberg School of Medicine, Chicago, Illinois; 7 Department of Obstetrics and Gynecology, University of Utah, Salt Lake City; 8 Department of Obstetrics and Gynecology, Brown University, Rhode Island; 9 Barbra Streisand Women’s Heart Center, Smidt Heart Institute, Cedars-Sinai Medical Center, Los Angeles, California; 10 Department of Obstetrics and Gynecology, Columbia University, New York, New York

## Abstract

This cohort study of US births examines the association of preconception diet quality with adverse pregnancy outcomes.

## Introduction

Adverse pregnancy outcomes (APOs) are a major cause of maternal and neonatal morbidity.^1^ Preconception diet quality is associated with APO risk,^2^ but prior pregnancy-related indices had notable limitations.^3^ We developed the Prepregnancy Dietary Quality Index (PreP-DQI) to assess its association with APOs and cardiometabolic health.

## Methods

This cohort study used data from the Nulliparous Pregnancy Outcomes Study: Monitoring Mothers-to-Be (NuMoM2b) and the NuMoM2b-Heart Health Study (NuMoM2b-HHS),^4,5^ excluding individuals who delivered before 23 weeks of gestation, had fetal anomalies, missing dietary data, or had implausible caloric intake (below 1st percentile or above 99th percentile). All participants in the original studies provided written informed consent. This analysis was exempted from institutional review board approval at Old Dominion University as the dataset is deidentified, and followed the Strengthening the Reporting of Observational Studies in Epidemiology (STROBE) reporting guideline.

Preconception diet was assessed using the Block/Bodnar 2010 food frequency questionnaire (FFQ), self-administered at 6 to 13 weeks of gestation during NuMoM2b enrollment (2010-2013). The PreP-DQI was developed using the 2020-2025 US Department of Agriculture (USDA) guidelines,^6^ which consisted of 23 adequacy and 4 moderation components, each scored from zero to 5. The final PreP-DQI was scored from zero to 100, with higher scores indicating healthier diets (eAppendix 1 in Supplement 1).

The primary outcome was a composite of APOs, defined as occurrence of 1 or more of the following: hypertensive disorders of pregnancy (HDP), preterm births, small for gestational age (SGA), stillbirth, or gestational diabetes (eAppendix 2 in Supplement 1). Secondary outcomes included first-trimester cardiometabolic biomarkers (eTable in Supplement 1).

Internal consistency of the PreP-DQI was assessed using Cronbach α. Individuals were divided into quartiles based on PreP-DQI scores (Q1 [least healthy diet] to Q4 [healthiest diet]). Energy-adjusted food group and nutrient intakes (per 1000 kcal) were assessed across quartiles using linear trend tests to evaluate diet quality and dose-response relationships.

Missing outcome and covariate data were handled by multiple imputations. Adjusted relative risks (aRRs) with 95% CIs were calculated using modified Poisson regression with robust variance, with Q1 as reference, adjusting for maternal age, body mass index, race (self-identified with categories including Asian, Hispanic, non-Hispanic Black, non-Hispanic White, and other), employment, marital status, education, insurance type, energy intake per day, exercise (MET-hrs) during the first trimester, and smoking before pregnancy. E-values were calculated for statistically significant associations to quantify unmeasured confounding.

In the HHS cohort, log-transformed biomarkers were modeled using linear regression, adjusting for the same covariates as the primary analysis, with adjusted *P* value obtained from quartile entered as an ordinal score. The threshold for significance was *P* < .05 in 2-sided tests. Statistical analyses were conducted using R version 4.4.2 (R Project for Statistical Computing).

## Results

A total of 7551 (75.2%) individuals from the nuMoM2b cohort met eligibility criteria for this analysis (mean [SD] age, 27.3 [5.5] years; 822 Black [10.9%], 1254 Hispanic [16.6%], 4795 White [63.5%]), including 3520 (46.6%) in the HHS cohort. Higher PreP-DQI quartiles were associated with significantly older age, lower BMI, White or Asian race, higher education, marriage, employment, commercial insurance, higher income, lower prevalence of smoking tobacco before pregnancy, greater total energy intake, and greater exercise during the first trimester.

The mean (SD) PreP-DQI score was 66.0 (9.4). The PreP-DQI score demonstrated high internal consistency among its component measures (Cronbach α = 0.93). Across PreP-DQI quartiles, intake of adequacy components increased significantly (except dairy and vitamin D), whereas intake of most moderation components decreased significantly (except sodium). Compared with individuals in Q1, individuals in Q3 had lower risk preterm birth (aRR, 0.76; 95% CI, 0.59-0.98; E-value: 1.96) and individuals in Q4 had lower risks of APOs (aRR, 0.77; 95% CI, 0.66-0.89; E-value, 1.92), preterm birth (aRR, 0.73; 95% CI, 0.55-0.97; E-value, 2.08), and HDP (aRR, 0.77; 95%CI, 0.62-0.95; E-value, 1.92) (Figure). In complete case analysis, compared with individuals in Q1, individuals in Q4 had lower risks of APO (aRR, 0.76; 95% CI, 0.65-0.89), preterm birth (aRR, 0.74; 95% CI, 0.55-1.00), and HDP (aRR, 0.77; 95% CI, 0.62-0.95).

**Figure.**
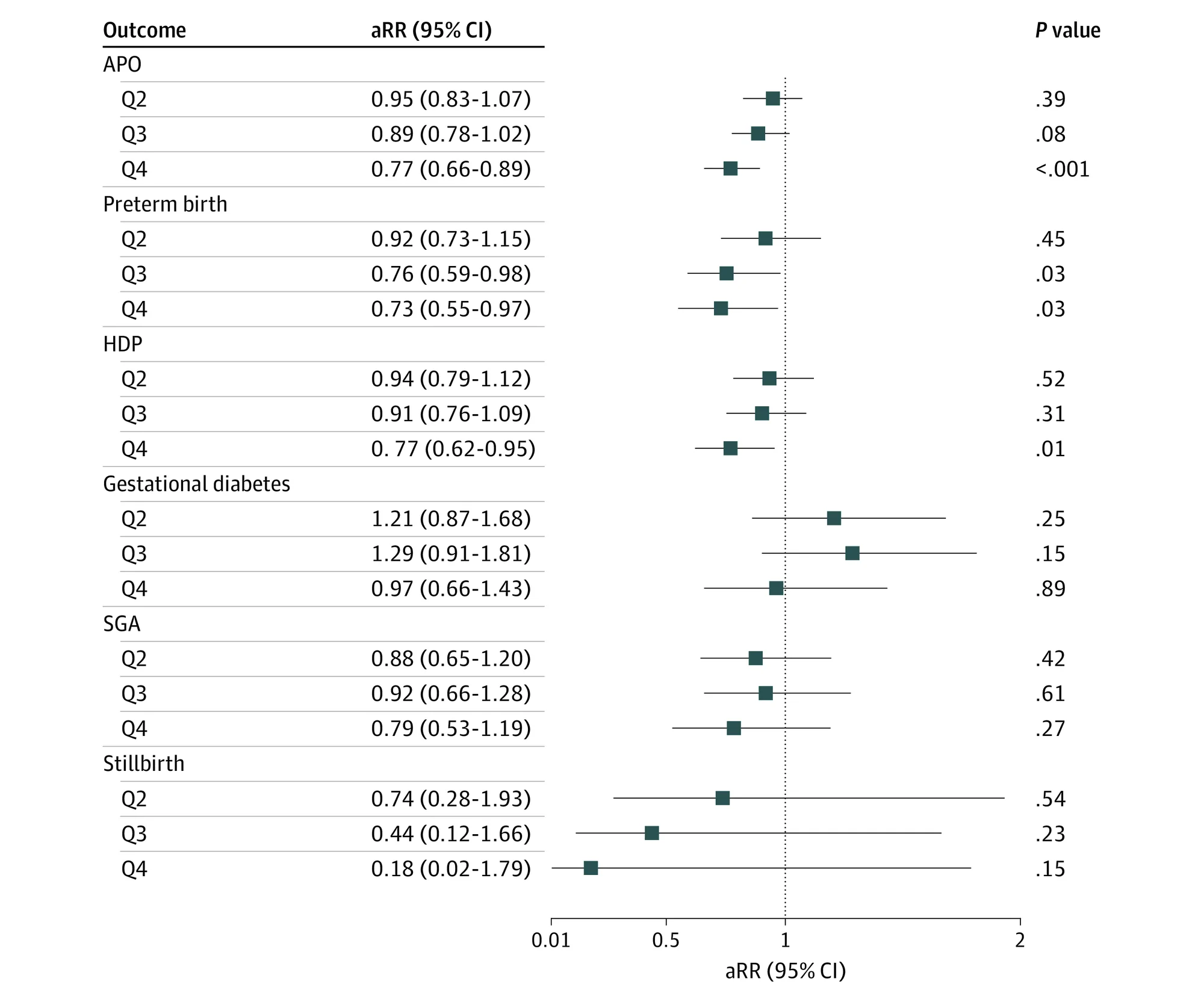
Association Between the Prepregnancy Dietary Quality Index and Adverse Pregnancy Outcomes (APOs) Using Multiple Imputation aRR indicates adjusted relative risk; HDP, hypertensive disorders of pregnancy; Q, quartile; SGA, small for gestational age. Quartiles are based on Prepregnancy Dietary Quality Index scores, with the first quartile serving as reference.

Across higher PreP-DQI quartiles, median (IQR) insulin levels decreased from 7 (5-13) to 5 (3-7) uIU/mL, triglyceride levels from 84 (61-117) to 74 (57-103) mg/dL, hs-CRP levels from 0.17 (0.06-0.73) to 0.11 (0.05-0.25) mg/dL, and HDL-C levels increased from 51 (44-60) to 58 (50-67) mg/dL (Table). A web-based application for calculating the PreP-DQI was developed.^7^

**Table.** First-Trimester Cardiometabolic Biomarkers Across PreP-DQI Quartiles

Biomarkers	Median (IQR)				*P* value for trend
	Q1 (n = 915)	Q2 (n = 858)	Q3 (n = 895)	Q4 (n = 852)	
Insulin, uIU/mL	7 (5-13)	6 (4-10)	5 (4-9)	5 (3-7)	<.001
Glucose, mg/dL	89 (82-96)	89 (83-96)	89 (83-96)	88 (82-94)	.01
Hemoglobin A_1c_, %	5.2 (5.0-5.4)	5.2 (5.0-5.4)	5.2 (4.9-5.4)	5.1 (4.9-5.4)	<.001
Triglyceride, mg/dL	84 (61-117)	82 (60-115)	78 (58-110)	74 (57-103)	<.001
Cholesterol, mg/dL	174 (153-200)	178 (155-205)	183 (159-206)	182 (160-205)	<.001
HDL-C, mg/dL	51 (44-60)	53 (46-62)	56 (48-66)	58 (50-67)	<.001
LDL-C, mg/dL	102 (84-125)	105 (86-127)	106 (85-127)	106 (84-126)	.11
hs-CRP, mg/dL	0.17 (0.06-0.73)	0.18 (0.07-0.70)	0.15 (0.06-0.40)	0.11 (0.05-0.25)	<.001

Abbreviations: HDL-C, high density lipoprotein cholesterol; hs-CRP, high sensitivity C-reactive protein; LDL-C, low density lipoprotein cholesterol; PreP-DQI, Prepregnancy Dietary Quality Index; Q, quartile.

SI conversion factor: To convert cholesterol to mmol/L, multiply by 0.0259; C-reactive protein to mg/L, multiply by 10; glucose to mmol/L, multiply by 0.0555; hemoglobin A_1C_ to proportion of total hemoglobin, multiply by 0.01; insulin to pmol/L, multiply by 6.945; triglyceride to mmol/L, multiply by 0.0113.

## Discussion

In this cohort of nulliparous individuals, higher PreP-DQI scores were associated with lower risks of APOs, particularly preterm birth and HDP, underscoring the importance of assessing the overall preconception diet quality using a composite measure that incorporates both dietary adequacy and moderation across whole foods and nutrients. Dietary intake was self-reported using a validated FFQ and may be subject to recall and reporting bias. The Block/Bodnar 2010 FFQ was the pregnancy-adapted instrument administered during nuMoM2b enrollment (2010-2013), predating the Modern Block 2014 FFQ. Also, there is a possibility of a potential selection bias, as individuals with missing dietary and biomarker data were excluded. Thus, further validation of PreP-DQI in more diverse populations is warranted. The association between higher PreP-DQI scores and a healthier first-trimester cardiometabolic profile provides a biologic plausibility for these findings, highlighting the role of preconception diet quality.

## Notes

Supplement 1. eAppendix 1. Description of maternal and neighborhood characteristics eAppendix 2. Definition of outcomes eTable. Components and scoring of Prepregnancy Diet Quality Index (PreP-DQI) eReferences.

Supplement 2. Data Sharing Statement
