# Supplementary material for: Prepregnancy Diet Quality Index and Adverse Pregnancy Outcomes
Source: JAMA Netw Open. 2026 Sep 10;9(9):e2633245. doi: 10.1001/jamanetworkopen.2026.33245 (PMC13563257; doi:10.1001/jamanetworkopen.2026.33245)
Supplement: Supplementary file 1 [file jamanetwopen-e2633245-s001.pdf]

Supplemental Online Content

Bharmal U, Saade G, Greenland P, et al. Prepregnancy diet quality index and adverse pregnancy outcomes. *JAMA Netw Open.* 2026;9(9):e2633245. doi:10.1001/jamanetworkopen.2026.33245

eAppendix 1. Description of maternal and neighborhood characteristics

eAppendix 2. Definition of outcomes

eTable 1. Components and scoring of Prepregnancy Diet Quality Index (PreP-DQI)

eReferences.

This supplemental material has been provided by the authors to give readers additional information about their work.

eAppendix 1. Development of the PreP-DQI score

© 2026 Bharmal U et al. *JAMA Network Open*.

The PreP-DQI was developed using U.S. Department of Agriculture 1,800-kcal/day nutrient targets as standardized cutoffs to align with recommendations for early gestation.[1] This level was selected to provide a consistent basis for assessing nutrient density, rather than reflecting participants' actual caloric requirements, which vary based on their pre-pregnancy BMI.

Food groups and nutrients were classified as adequacy components (higher intake beneficial; n = 23, including vegetables, fruits, whole grains, animal and plant based proteins, oils, and key micronutrients including folate, iron, calcium, vitamin D, and essential fatty acids) or moderation components (lower intake beneficial; n = 4, including added sugars, saturated fat, sodium, and refined grains) (eTable 1). Each component was scored from 0 to 5. For adequacy components, higher intakes received higher scores (5 for meeting or exceeding the recommended minimum, 0 for no intake), while for moderation components, higher scores reflected lower intakes (5 for meeting or staying below the recommended maximum, 0 if exceeding HEI-2015 cutoffs), with the scores scaled proportionally between minimum and recommended intakes. All recommended minimum and maximum intakes were based on the 2020-2025 USDA dietary guidelines, while HEI-2015 cutoffs were only used to define the threshold for 0 points in moderation components.[1,2] To reflect their relative contributions to overall diet quality, adequacy and moderation domains were weighted 60% and 40%, respectively, consistent with the HEI-2015 framework.[2] The adequacy domain (23 components; maximum raw score = 115) and moderation domain (4 components; maximum raw score = 20) were separately rescaled to contribute maximum scores of 60 and 40 points, respectively. The weighted domain scores were then summed to generate the final PreP-DQI score, ranging from 0 to 100. The final PreP-DQI score (0–100) reflects overall preconception diet quality, with higher scores indicating healthier diets.

© 2026 Bharmal U et al. *JAMA Network Open*.

eAppendix 2. Definition of outcomes

The primary outcome was a composite of APOs, including HDP (gestational hypertension, preeclampsia, HELLP syndrome [hemolysis, elevated liver enzymes, low platelet count], or eclampsia), PTB (less than 37 weeks 0 days), SGA (birth weight <5 th percentile), stillbirth, and GDM.

Gestational hypertension was defined as the occurrence of new-onset hypertension, defined as systolic blood pressure 140 mmHg or greater or diastolic blood pressure 90 mmHg or greater on 2 occasions at least 6 hours apart or 1 occasion with subsequent antihypertensive therapy, excluding blood pressures recorded during the second stage of labor. Pre-eclampsia was defined as new-onset hypertension (systolic blood pressure ≥140 mmHg or diastolic blood pressure ≥90 mmHg) after 20 weeks of gestation in individuals with previously normal blood pressure, accompanied by proteinuria (≥300 mg/day, urine protein/creatinine ratio ≥0.3). Eclampsia was defined as the occurrence of a seizure without a known cause other than a hypertensive disorder of pregnancy.[3] Based on Alexander's fetal growth curves, SGA was defined as birthweight less than the 5th percentile for gestational age.[4] Stillbirth will be defined as fetal death at 20 weeks or greater. GDM will be defined by one of the following glucose tolerance test (GTT) criteria: 1) 100-gram GTT (fasting 3-hour) with two abnormal values (fasting ≥95 mg/dL, 1-hour ≥180 mg/dL, 2-hour ≥155 mg/dL, or 3-hour ≥140 mg/dL); 2) 75-gram GTT (fasting 2-hour) with one abnormal value (fasting ≥92 mg/dL, 1-hour ≥180 mg/dL, 2-hour ≥153 mg/dL; or 3) 50-gram: GTT ≥200 mg/dL (no fasting 1-hour).[5]

Secondary outcomes included first-trimester cardiometabolic biomarkers, including insulin, glucose, hemoglobin A1c (HbA1c), triglycerides, total cholesterol, high-densitylipoprotein cholesterol (HDL-C), low-density lipoprotein cholesterol (LDL-C), and high-sensitivity C-reactive protein (hs-CRP). Non-fasting maternal serum (from 4-8 mL of blood) samples collected during the first pregnancy visit (6-13 weeks' gestation) of the nuMoM2b study.[6–8] For participants who participated in the nuMoM2b-HHS cohort, the serum was assayed for cardiometabolic biomarkers.

© 2026 Bharmal U et al. *JAMA Network Open*.

eTable 1. Components and Scoring of Pre-Pregnancy Diet Quality Index (PreP-DQI)

Score components	Criterion for maximum score of “5”	Criterion for minimum score of “0”
Adequacy components		
Total vegetables (cup eq./day)	≥ 2.5	No intake
Dark green vegetables (cup eq./week)	≥ 1.5	No intake
Red and yellow vegetables (cup eq./week)	≥ 5.5	No intake
Starchy vegetables (cup eq./week)	≥ 5	No intake
Beans, peas, and lentils (cup eq./week)	≥ 1.5	No intake
Other vegetables (cup eq./week)	≥ 4	No intake
Fruits (cup eq./day)	≥ 1.5	No intake
Whole grains (ounce eq./day)	≥ 3	No intake
Animal-based protein (ounce eq./week)	≥ 23	No intake
Plant-based protein sources (ounce eq./week)	≥ 4	No intake
Dairy (cup eq./day)	≥ 3	No intake
Seafood (ounce eq./week)	≥ 8	No intake
Oils (grams/day)	≥ 24	No intake
Calcium (milligrams/day)	≥ 1000	No intake
Folate (micrograms/day)	≥ 600	No intake
Iron (milligrams/day)	≥ 27	No intake
Magnesium (milligrams/day)	≥ 360	No intake
Fiber (grams/day)	≥ 25	No intake
Linoleic acid (grams/day)	≥ 13	No intake
Alpha-Linoleic acid (grams/day)	≥ 1.4	No intake
Choline (milligrams/day)	≥ 450	No intake
Vitamin B12 (micrograms/day)	≥ 2.6	No intake
Vitamin D (IU/day)	≥ 600	No intake
Moderation components		
Added sugars (% of total kcal/day)	≤ 10	≥ 26
Refined grains (ounce eq./day)	≤ 3	≥ 7.7
Saturated fats (% of total kcal/day)	≤ 10	≥ 16

© 2026 Bharmal U et al. *JAMA Network Open*.

Sodium (milligrams/day)	≤ 2300	≥ 3900

© 2026 Bharmal U et al. *JAMA Network Open*.

eReferences.

1. Snetselaar LG, de Jesus JM, DeSilva DM, Stoody EE. Dietary Guidelines for Americans, 2020–2025. *Nutr Today*. 2021;56(6):287-295. doi:10.1097/NT.0000000000000512

2. Krebs-Smith SM, Pannucci TE, Subar AF, et al. Update of the Healthy Eating Index: HEI-2015. *J Acad Nutr Diet*. 2018;118(9):1591-1602. doi:10.1016/j.jand.2018.05.021

3. Gestational Hypertension and Preeclampsia: ACOG Practice Bulletin, Number 222. *Obstet Gynecol*. 2020;135(6):e237-e260. doi:10.1097/AOG.0000000000003891

4. Alexander G, Himes J, Kaufman R, Mor J, Kogan M. A united states national reference for fetal growth. *Obstet Gynecol*. 1996;87(2):163-168. doi:10.1016/0029-7844(95)00386-X

5. Gestational Diabetes Mellitus | ACOG. Accessed October 4, 2024. https://www.acog.org/clinical/clinical-guidance/practice-bulletin/articles/2018/02/gestational-diabetes-mellitus

6. Haas DM, Parker CB, Wing DA, et al. A description of the methods of the Nulliparous Pregnancy Outcomes Study: monitoring mothers-to-be (nuMoM2b). *Am J Obstet Gynecol*. 2015;212(4):539.e1-539.e24. doi:10.1016/j.ajog.2015.01.019

7. Haas DM, Ehrenthal DB, Koch MA, et al. Pregnancy as a Window to Future Cardiovascular Health: Design and Implementation of the nuMoM2b Heart Health Study. *Am J Epidemiol*. 2016;183(6):519-530. doi:10.1093/aje/kwv309

8. Jancsura MK, Wirth MD, Helsabeck NP, et al. Dietary Inflammatory Index Is Differentially Associated With Cardiometabolic Health After Pregnancy on the Basis of Adverse Pregnancy Outcome Exposure. *J Am Heart Assoc*. 2024;13(24):e035391. doi:10.1161/JAHA.124.035391

© 2026 Bharmal U et al. *JAMA Network Open*.
