# Supplementary material for: Prepregnancy Diet Quality Index and Adverse Pregnancy Outcomes
Source: JAMA Netw Open. 2026 Sep 10;9(9):e2633245. doi: 10.1001/jamanetworkopen.2026.33245 (PMC13563257; doi:10.1001/jamanetworkopen.2026.33245)
Supplement: Supplementary file 2 [file jamanetwopen-e2633245-s002.pdf]

Data Sharing Statement

Bharmal. Prepregnancy Diet Quality Index and Adverse Pregnancy Outcomes. *JAMA Netw Open*. Published September 10, 2026. doi:10.1001/jamanetworkopen.2026.33245

Data
Data available: Yes
Data types: Deidentified participant data
How to access data: Data are available at NICHD DASH.
When available: With publication

Supporting Documents
Document types: None

Additional Information
Who can access the data: Researchers whose proposed use of the data has been approved
Types of analyses: Research purpose.
Mechanisms of data availability: after approval of a proposal and with a signed data access agreement
